# Antioxidant Levels in Cord Blood of Term Neonates and Its Association with Birth Weight

**Published:** 2016

**Authors:** Mehrdad MIRZARAHIMI, Adel AHADI, Shahab BOHLOOLI, Esmaeil NAMAKIKHALAJAN, Manouchehr BARAK

**Affiliations:** 1 Department of Pediatrics, Faculty of Medicine, Ardabil University of Medical Science, Ardabil, Iran; 2 Drug and Advanced Sciences Research Center, School of Pharmacy, Ardabil University of Medical Science, Ardabil, Iran; 3 General Practitioner, Ardabil University of Medical Science, Ardabil, Iran

**Keywords:** Antioxidants, Catalase, Term newborn, Cord blood, Birth weight

## Abstract

**Objective:**

Due to excessive production of free radicals and antioxidants evolved mechanisms against oxidative stress, infants are very vulnerable. As there was a significant relation between antioxidant levels and birth weight, we aimed verify this relationship.

**Materials & Methods:**

In this descriptive analytical study we evaluated the antioxidant status of 40 healthy term newborns (gestation age 38-42 wk) with weight >2500 g (AGA) and 40 healthy term newborns (gestation age 38-42 wk) with LBW babies (weight < 2500 g) (SGA) in Ardabil Buali Hospital, Ardabil, northwest Iran in 2014. About 15 Ml of cord blood was collected after the second stage of labor. The levels of vitamin A, E, and C, catalase, glutathione peroxidase (GPX), bilirubin and serum uric acid were measured by standard methods. Informed consent was obtained from newborn mothers and study protocol was approved by university Ethics Committee. Data were analyzed using SPSS.19.

**Results:**

The mean levels of bilirubin, vitamin C, E, catalase and GPX in AGA group were significantly higher than SGA group but the mean of serum uric acid in SGA group was more than AGA. In addition, the mean of vitamin A was similar in two groups. There was a significant relation between antioxidant levels and birth weight in term newborns.

**Conclusion:**

In line with other studies the amounts of antioxidant levels except serum uric acid in AGA group was significantly more than SGA group.

## Introduction

Human life always has been threatened by oxidative stress caused by free radicals and reactive oxygen groups. Human body has antioxidant defense dams, which have important role against oxidative stress. Preventive antioxidants in the first line of defense prevent the formation of free radicals and reactive oxygen groups. In the third barrier, body antioxidant enzymes (such as superoxide dismutase, catalase, glutathione peroxidase, etc.) maintain biological macromolecules against oxidative stress. Some health problems are leading causes of maternal and perinatal mortality, morbidity, and lead to low birth weight (LBW) and prematurity that cause decreasing the level of antioxidant status in mothers (1). In fact, oxidative stress damages living systems by a variety of active oxygen radicals produced in excess of the body’s antioxidant defense. All diseases are involved with free radicals. In many cases, free radicals can cause disease process secondarily, whereas in some cases, free radicals are produced because of the disease. Therefore, the narrow balance is between oxidants and antioxidants in health and disease. The level of vitamin A in the LBW babies is significantly lower in cord blood (2). Oxidative stress normally is higher during pregnancy than other times due to increased oxygen consumption and energy needs. Due to excessive production of free radicals and antioxidants evolved mechanisms against oxidative stress, infants are very vulnerable. There was a significant relation between antioxidant levels and LBW, because low levels of antioxidants in the newborns, make them vulnerable to oxidative stress and increased mortality and morbidity of them (2,3). The aim of this study was to evaluate antioxidant levels in cord blood related to birth weight in term newborns.

## Materials & Methods

In this descriptive analytical study, we evaluated the antioxidant status of 40 healthy term newborns (gestation age 38-42 wk) with weight >2500 g (AGA) and 40 healthy term newborns (gestation age 38-42 wk) with LBW babies (weight < 2500 g) (SGA) in Ardabil Buali Hospital, Ardabil, northwestern Iran in 2014. About 15 Ml of cord blood was collected after the second stage of labor. The levels of vitamin A, E, and C, catalase, glutathione peroxidase (GPx), bilirubin and serum uric acid were measured by standard methods. We used catalase kits, ELISA and auto analyzer to measure the activity of catalase, method of Paglia and Valentine (4) for measuring the activity of GPx, and HPLC method for measurement of vitamin. Exclusion criteria were perinatal asphyxia, infants of mothers with diabetes and hypertension, infants with metabolic disease and congenital malformations. Informed consent was obtained from newborn mothers and study protocol was approved by Ethics Committee of Ardabil University of Medical Science, Ardabil, Iran. Data were analyzed using SPSS.19 (Chicago, IL, USA) and the significant level set at P<0.05.

## Results

Fifty percent of SGA newborns and 42.5% of AGA newborns had bilirubin in normal range (1-0.5 mg/dl) and the mean of bilirubin in AGA group was significantly more than SGA (P=0.01). Of all AGA group three, 7.5% and of SGA group two, 5% of newborns had G6PD deficiency but it was not statistically significant. The levels of vitamin C, vitamin E, catalase, GPx in AGA group were significantly higher than SGA groups (P=0.039). In addition, the level of vitamin A was not significant between two groups but the level of serum uric acid in AGA group was significantly lower than SGA group (P=0.024) (Table 1).

**Table 1:** Levels of Various Antioxidants in Study Infants

**Groups**	**Birth weight**	**Vitamin A**	**Vitamin E**	**Vitamin C**	**Catalase**	**Glutathione peroxidase**	**Serum uric acid**	**Bilirubin**
**AGA**	>2500 g	30.4±6.2	0.6±0.2	1.4±0.5	98.8±16.4	51.4±11.8	4.1±1.3	1.1±0.4
**SGA**	<2500 g	28.7±5.9	0.5±0.2	1.2±0.5	90.4±15.4	45.9±11.6	5.2±1.7	0.9±0.4
***P*** ** value**		0.22	0.047	0.041	0.019	0.039	0.024	0.01

## Discussion

In this study, the levels of vitamin C, vitamin E, catalase, GPx in AGA group were significantly higher than SGA group. The serum uric acid in SGA group was higher than AGA group (P=0.024) and the difference of level of vitamin A and G6PD was not significant between two groups. In a study, the level of vitamin A decreased with reduction of weight, the level of vitamin E increased with the increase of weight, and there was direct and significant relationship between newborn weight and catalase and super dismutase (3). LBW newborns were deficient in several important antioxidants, which may predispose them to higher oxidative stress (3). The level of super oxide dismutase in SGA newborns was more than AGA but this finding was not significant, besides, the level of catalase, G6PD enzyme activity and vitamin E in SGA group was significantly more than AGA group (4). The levels of GPx, NADPH proportion and vitamin A in AGA group were significantly more than SGA group (5). In hyper bilirubin newborns the levels of vitamins C, E was significantly lower than other newborns (6). The level of peroxidase in SGA group was significantly higher than AGA and the tolerance of antioxidant in SGA newborns was significantly lower than AGA (7). In LBW newborns the levels of peroxidant lipids and oxidant proteins increased but the levels of vitamins A, E and C decreased (8). In our study, the serum uric acid in SGA group was significantly more than AGA group, which was similar to Akahori et al. study, where the results revealed that the uric acid could be an independent risk factor for SGA (9). In the present study, for the probably influence of maternal Body Mass Index (BMI) on antioxidant status, we analyzed the relation between antioxidant levels and mothers BMI by statistical tests. Accordingly, there was not any significant relationship between the levels of bilirubin, serum uric acid, vitamin A, C, E, catalase and Glutathione and mother BMI.

**In conclusion**, the levels of vitamin A, C and E in our study are in agreement with other studies and, in line with other studies the amounts of antioxidant levels except serum uric acid in AGA group was significantly more than SGA group.

## References

[1] Howlader MZ1, Parveen S, Tamanna S, Khan TA, Begum F (2009 ). Oxidative stress and antioxidant status in neonates born to pre-eclamptic mother. J Trop Pediatr.

[2] Adhikari SC, Somani BL, Kalra MS, Mathai SC, Arora BMM (2011). Umbilical cord blood plasma vitamin A levels in low birth weight (LBW) babies. MJAFI.

[3] Kumar A, Ranjan R, Basu S, Khanna HD, Bhargava V (2008). Antioxidant levels in cord blood of low birth weight newborns. Indian Pediatr.

[4] Paglia DE, Valentine WN (1967). Studies on the quantitative and qualitative characterization of erythrocyte glutathione peroxidase. J Lab Clin Med.

[5] Lee Y, Chou Y (2005). Antioxidant Profiles in Full Term and Preterm Neonates. Chang Gung Med J Des.

[6] Abdul-Razzak KK, Almomany EM, Nusier MK, Obediat AD, Salim AM (2008). Antioxidant vitamins and glucose-6-phosphate dehydrogenase deficiency in fullterm neonates. Ger Med Sci.

[7] Gveric-Ahmetasevic S, Sunjic SB, Skala H, Andrisic L, Stroser M, Zarkovic K, Skrablin S, Tatzber F, Cipak A, Jaganjac M, Waeg G, Gveric T, Zarkovic N (2009). Oxidative stress in small-for-gestational age (SGA) term newborns and their mothers. Free Radic Res.

[8] Negi R, Pande D, Kumar A, Khanna RS, Khanna HD (2012). Evaluation of biomarkers of oxidative stress and antioxidant capacity in the cord blood of preterm low birth weight neonates. J Matern Fetal Neonatal Med.

[9] Akahori Y, Masuyama H, Hiramatsu Y (2012). The correlation of maternal uric acid concentration with small-forgestational- age fetuses in normotensive pregnant women. Gynecol Obstet Invest.

